# Experimental study of non-oxidized and oxidized bitumen obtained from heavy oil

**DOI:** 10.1038/s41598-021-87398-2

**Published:** 2021-04-14

**Authors:** Richard Djimasbe, Eduard A. Galiullin, Mikhail A. Varfolomeev, Revo Z. Fakhrutdinov, Ameen A. Al-Muntaser, Abdolreza Farhadian

**Affiliations:** 1 Department of Petroleum Engineering, Kazan Federal University, Kremlevskaya Str. 18, Kazan, Russian Federation 420008; 2 Faculty of Petroleum and Petrochemistry, Kazan National Research Technological University, Karl Marx Str., 68, Kazan, Russian Federation 420015

**Keywords:** Chemistry, Energy science and technology, Engineering

## Abstract

Heavy oil and vacuum residue were used to obtain road bitumen BND 50/70 using two different methods of steam distillation at 323–362 °C and by oxidation, a method using packed column at temperature of 211–220 °C. The obtained residues using two methods steam distillation and oxidation are known as non-oxidized bitumen and oxidized bitumen, respectively. The products were evaluated using different standards including GOST 33133-2014, GOST 22245-90, and ASTM D5. The results showed that the yield of oxidized bitumen reached a maximal rate of 89.59% wt., while that of non-oxidized bitumen is 55% wt. The softening point of oxidized bitumen is 49–57 °C compared to non-oxidized bitumen (46–49 °C). Remarkably, the previous softening point and penetrability of 47–71 points of oxidized bitumen are consistent with norms to BND 50/70 bitumen, according standard. The non-oxidized bitumen has a relatively low softening point and a higher penetration value of 71–275, which refers to BND 200/300 bitumen. Comparatively, the use of a packed column is beneficial than the steam distillation, due to high capability of the nozzles to strengthens contact between feedstock and compressed air in the reaction zone and decreases the reaction time to 4.15 h.

## Introduction

Daily needs, due to the worldwide economic growth, increase road transport activities destroying the roads^1^. However, the necessity to improve the building and reconstructing of new roads became the main challenge of bitumen producers^2^. Given the gradual disappearance of conventional oil added to that, the petroleum industries are faced with a major challenge which is to develop new but less expensive techniques that facilitate bitumen production in order to remedy road infrastructure problems^3, 4^.

Nowadays, unconventional hydrocarbons sources such as heavy oil, natural bitumen, shale oil were discovered in many countries as Canada, Venezuela, Brasilia, Russia, Chad, and Madagascar^5–10^. The discovery of these resources will promote the growth of production in order to balance the energy deficit and that of bitumen in the world market^11^. By definition, heavy oil and natural bitumen are characterized by high viscosity and low API density with a very low concentration of volatile distillate fractions, such as gasoil and kerosene, as well as high amount of resins and asphaltenes^12–16^.

Generally, the road bitumen is essentially assessed by the technology of production, the relative concentration of resins, and asphaltene. The respective components in the road bitumen play a particular role giving a synergistic performance to the bitumen. Asphaltene is the heaviest and responsible components for a non-newtonian character of bitumen, and they have a direct influence on the softening point and the rigidity of the bitumen. The resins promote good adhesion of bitumen to the surface of the minerals and of ductility (elasticity). Saturates and aromatics are responsible for the rheological property of the bitumen, such as viscosity^12, 13^.

To meet the challenges associated with the demands and requirements of the quality of road bitumen on the market, the development of new efficient techniques is necessary^17^. Among the methods already known are vacuum distillation of oil, air oxidation, a compound of heavy residues after vacuum distillation, selective extraction, and the residue from the tar deasphalting process^18–20^. Several series of works on the production of road bitumen have been carried out to obtain a better quality of bitumen adaptable to practical requirements as well as to point out the mechanism of the reactions which can occur during the oxidation of the vacuum residue and the upgrading of heavy oil. Abdullin et al. studied the thermal-oxidative stability of petroleum bitumen using «over-oxidation» from 240 to 260 °C with the oxidation time from 4 to 8 h. They reported that the softening temperature increased from 30.5–36 °C as a function of the oxidation time. The properties such as viscosity-temperature and the ductility at 0 °C have decreased^21^. Hilde Soenen et al. experimented the oxidation process of two different residues from the visbreaking process and cracking process using the laboratory air-blowing vessel at 260 °C, and airflow of 1 l/min, under atmospheric pressure. They concluded the needle penetration of bitumen is 187–190 points and the softening point 36.9 °C and 39.2 °C according to EN 1426 and EN 1427^22^. Chaala et al. carried out vacuum pyrolysis of automobile shredder residues using the pyrolytic oil as a modifier for road bitumen, and they indicated that the needle penetration of bitumens is 113 and 204, the softening point was 45 °C and 45.8 °C and Fraass point were − 7 °C and − 8.5 °C^23^. They also concluded that, mixing the vacuum bitumen and the pyrolytic residue can decrease the penetrability and increase the softening point due to the compositional change of bitumen then these bitumen can behave as non-Newtonian fluids with high viscosity^24^. On the side of non-oxidized bitumens, few works and literature were presented, except Chaale et al. according to its physicochemical and rheological analyzes of residue from vacuum pyrolysis, they declared that the pyrolytic residue is considered to be petroleum bitumen^23^.

In the above mentioned studies, authors proposed different methods of producing road bitumen taking into account all parameters such as temperature, airflow, pressure, time, and raw materials^25–27^**,** except to improve equipment performances including column and accessory, to reduce the cost of production**.** Secondly, no comparison was done on the obtained products by different methods in the mentioned works. However, this work focuses on using a specially packed oxidation column to increase the contact surface between the feedstock and the injected compressed air to reduce the production cost, followed by comparison of the oxidized with non-oxidized bitumen obtained by the steam distillation**.** For evaluation the performance of obtained products various method such as penetration of the needle into the bitumen to determine the mark and rigidity of the bitumen by the penetrometer are essential. In addition, the softening temperature measured by the ball and ring method to determine the maximum bitumen operating temperature, ductility is determined by a ductilometer set at 0 °C, brittleness temperature is obtained by the Fraass method^20^ the rheological measurements (viscosity and adhesion capability of bitumen) as well as determining the composition of the bitumen by SARA (saturates, aromatics, resins, and asphaltenes) analysis.

## Results and discussions

### Steam distillation process (production of non-oxidized bitumen)

Tables 1 and 2 show the results of the obtained products after steam distillation of heavy oil. The results reveal that the increase of temperature of the bottom (302–340 °C ± 2 °C) and top (150–201 °C ± 1 °C) of the column decreases the yield of bitumen from 55% ± 0.3 to 47% ± 0.2 respectively. On the other hand, this rise in temperatures decreases the penetration of bitumen from 275 to 71 units. A decrease in bitumen yield occurred due to reactions as evaporation and condensation of high molecular components (resins and asphaltenes). The yield of bitumen and its properties are mostly promoted by the bottom temperature of the column and determined by the temperature of the superheated water steam. Remarkably, when the temperature at the bottom part of the column is around 300 °C, the bottom collected product (non-oxidized bitumen) seems to be as viscous plastic whiles with an increasing temperature to 340 °C and more, the non-oxidized bitumen residue presents a low penetration until 71 units. Thus, varying the bottom temperature of the column helps obtain the required bitumen residue with different penetration. Therefore, it should be mentioned that the expected bitumen properties depend mostly on the temperature of the bottom part of column, preferably above 300 °C. The temperature remains a key factor for the process of steam distillation of heavy oil for the production of the non-oxidized bitumen. Parallelly, the light hydrocarbons (synthetic oils) are the resulting condensation of the steam of light hydrocarbons; they have been obtained by the side of the column. The result showed that the yield of the light synthetic oil I increased in samples 1 and 2 from 34 to 37% and decreased to 23% in sample 3. The synthetic oil II exponentially increased from 11 to 14% and up to 30% respectively in samples 1, 2, and 3, with an increase in temperature. This explains why the rate of light fractions in the heavy oil is less than the middle fractions. The top temperature of the column allows controlling the amount of the top products while the bottom temperature of the column ensures the control of the amount and quality of the non-oxidized bitumen.

**Table 1:** Experimental parameters of steam distillation of heavy oil for non-oxidized bitumen.

Samples	Time, min	Top column’s temperature °C	Bottom column’s temperature, °C	Oil pre-heater temperature, °C	Bitumen yield, %	Penetration at 25 °C, mm
1	30	151 ± 1	302 ± 2	300 ± 2	55 ± 0.3	275 ± 0.1
2	35	187 ± 1	330 ± 2		49 ± 0.1	101 ± 0.1
3	40	201 ± 1	340 ± 2		47 ± 0.2	71 ± 0.1

**Table 2:** Yield of products of synthetic oils after steam distillation of heavy oil.

Products	1		2		3	
	Weight, g	%	Weight, g	%	Weight, g	%
1. Synthetic oil I	1716 ± 0.1	34 ± 0.1	952 ± 0.1	37 ± 0.4	869 ± 0.1	23 ± 0.3
2. Synthetic oil II	770 ± 0.1	11 ± 0.3	506 ± 0.1	14 ± 0.5	1138 ± 0.1	30 ± 0.2
3. Non-oxidized bitumen	1630 ± 0.1	55 ± 0.3	917 ± 0.1	49 ± 0.3	1806 ± 0.1	47 ± 0.2

### Synthetic oil properties analysis after steam thermal upgrading

Table 3 presents the results of the synthetic oil's properties. The generation of gases occurred through thermal cracking of C-S bonds and possibly other hydrocarbons bonds. Therefore, due to the distillation, the thermal cracking and the partial removal of sulfur would be the reasons for the viscosity decrease of heavy oil from 2268 mm^2^/s to that of synthetic oils of 16.5 mm^2^/s thereby, the density of synthetic oils I and II slightly increased from 892 to 915 kg/m^3^. The partially removed sulfur content is decreased around from 4.37% to 2.84% ± 0.01. During steam distillation process of heavy oil, possible thermal cracking reaction, physical distillation (separation), hydrocarbons cracking, and the condensation of high molecular components can occur by C-S destruction resulting into sulfur decrease in the product: (Eq. 1).

1 $${\text{R}}{-\!\!-}{\text{CH}}_{{2}} {-\!\!-}{\text{CH}}_{{2}} {-\!\!-}{\text{CH}}_{{2}} {-\!\!-}{\text{S}}{-\!\!-}{\text{CH}}_{{3}} + {\text{ 2H}}_{{2}} {\text{O }} \to {\text{ R}}{-\!\!-}{\text{CH}}_{{2}} {-\!\!-}{\text{CH}}_{{2}} {-\!\!-}{\text{CH}}_{{2}} {-\!\!-}{\text{CH}}_{{3}} + {\text{ H}}_{{2}} {\text{S }} + {\text{ H}}_{{2}} {\text{O}}$$

**Table 3:** Parameters of raw materials and synthetic oils.

No	Parameter	Initial heavy oil	Products of reaction (synthetic oil)		
			1	2	3
1	Sulfur content, % wt	4.2 ± 0.1	2.8 ± 0.2	3.7 ± 0.2	3.6 ± 0.1
2	Density at 20° C, kg/m^3^	963 ± 0.1	892 ± 0.1	919 ± 0.1	915 ± 0.1
3	The yield of fractions, % wt				
	200 °C 300 °C 350 °C	4 ± 0.6 12 ± 0.2 20 ± 0.1	8 ± 0.3 37 ± 0.1 52 ± 0.1	4 ± 0.6 18 ± 0.2 24 ± 0.1	– – –
4	Viscosity at 20° C, cSt (mm^2^/s)	2268 ± 0.1	16.4 ± 0.1	131 ± 0.1	55.7 ± 0.1

### Analysis of obtained oxidized bitumen

Table 4 illustrates the results of the material balance for the oxidation process of vacuum residue. The results show that sample 1 with oxidation time of 3 h and the obtained bitumen has a penetration of 71 points, which refers to bitumen BND 70/100 according to Russian standard GOST 33133-2014. For sample 2, obtained with increasing of oxidation time to 3.15 h, the penetration value continues to decrease to 65 points and refers to bitumen BND 50/70 according to the previous norm of GOST 33133-2014. So, by increasing oxidation time until 4.15 h, sample 3 of the obtained bitumen decreases in penetration value up to 47 units which analogically refers to bitumen BND 35/50, according to GOST 33133-2014. The penetration values of the obtained bitumens respectively BND 70/100; BND 50/70; and BND 35/50, characterize the mark and stiffness of bitumens, and these are proportional to the rate of resins and asphaltenes in the bitumens, as well as the penetrability of the needle in the bitumen, referred to the standard of GOST 33133, ASTM D5, and EN-12591:2010. Note that the properties and the production of the oxidized bitumen do not only depend on temperature, as observed in the non-oxidized bitumen, but it also depends on the time and equipment. The aim of this work has been reached; the oxidized bitumen BND 50/70 was obtained at 220 °C ± 1 °C and with time around 3.15–4.15 h.

**Table 4:** Results of the oxidized bitumen after oxidation process.

Samples	Time, hours	Temperature top column, °C	Temperature middle of the column, °C	Temperature Bottom columns, °C	Penetration at 2 °C, 0.1 mm
1	3.00	162 ± 2	216 ± 2	213 ± 2	71
2	3.15	163 ± 2	220 ± 2	221 ± 2	65
3	4.15	154 ± 2	206 ± 2	197 ± 2	47

### Properties of oxidized and non-oxidized bitumen

Table 5 shows the comparison results between the obtained bitumens by previously described methods. The results show that, non-oxidized bitumen has lower performance properties than oxidized bitumen according to the requirements of the standard (GOST 33133). For the samples of non-oxidized bitumens, only sample 3 has a penetration value of 71 units at 25 °C which refers to the bitumen 70/100 with softening point of 49 °C, and brittleness point of − 11 °C. Unfortunately, for the non-oxidized bitumens, despite the consistency in penetration for sample 3, the overall results are not consistent with the standard of GOST 33133-2014, ASTM, and D5 EN-12591:2010. The oxidized bitumens present good results, including samples 2 and 3 which have the penetration value at (47 to 71) at 25 °C and (23 to 30) at 0 °C. Their softening point is 49–57 °C, their brittleness are − 25 to − 29 °C (in opposite to the previous values of non-oxidized bitumen), and ductility from 1.5 to 3.5 cm. So, we conclude that the use of the specific packed column in this study is efficient and helped to soften the experimental conditions by decreasing the oxidation time to 3.15–4.15 h and temperature 220 °C, compared to traditional oxidation requiring more time of about 8 h and high a temperature of 260 °C^22^.

**Table 5:** Results of the properties of the obtained non-oxidized and oxidized bitumen.

Samples	Technology	Bitumen class	Penetration values		Temperature, 25 °C ± 1 °C		Ductility at 0 °C, 0.1 cm
			At 25 °C	At 0 °C	Softening	Brittleness	
1	Non-oxidized bitumen	200/300	275	–	39	− 20	0.3
2		100/130	101	–	46	− 13	0.2
3		70/100	71	–	49	− 11	0.2
1	Oxidized bitumen	70/100	71	30	49	− 25	3.5
2		50/70	65	30	51	− 29	3.3
3		35/50	47	23	57	− 25	1.5

### Determination of the group compositions of bitumens

**Figure 1:**
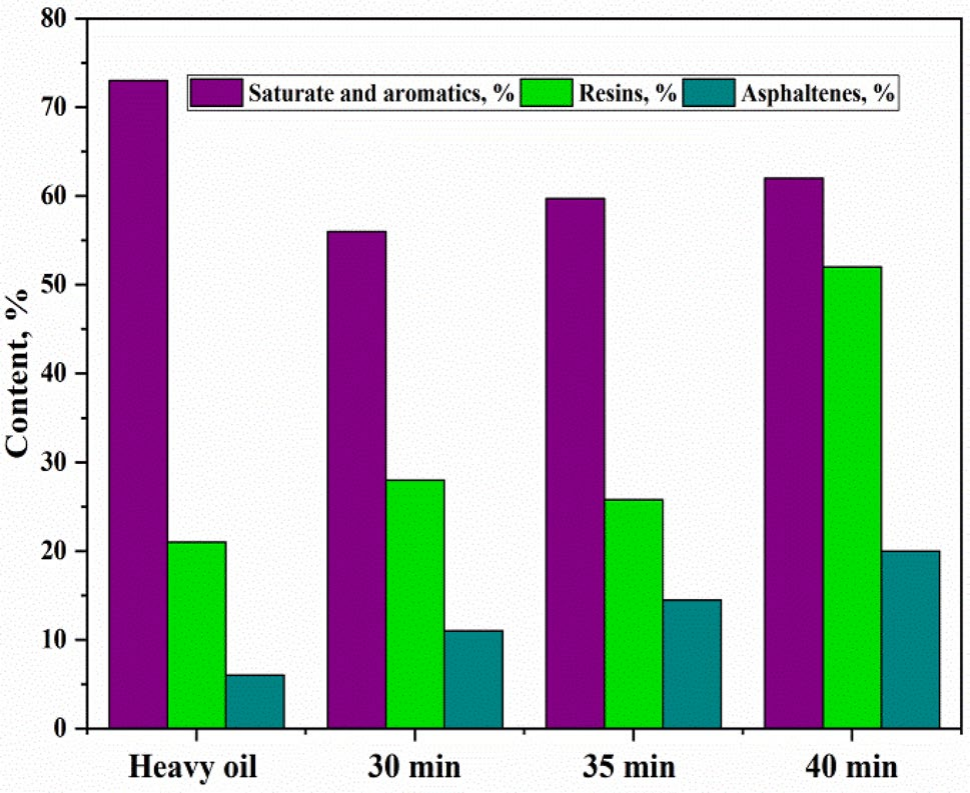
The schematic of composition fraction of feedstocks and of the non-oxidized bitumen.

**Figure 2:**
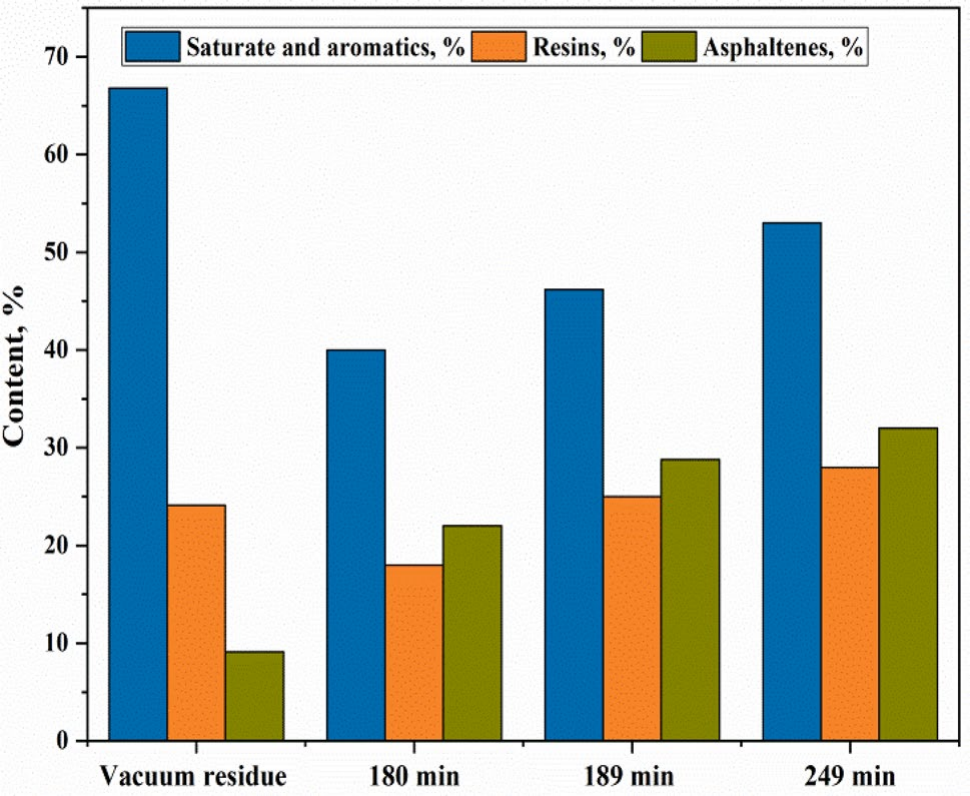
The schematic of composition fraction of feedstocks and of the oxidized bitumen.

Figures 1 and 2 present the results of the composition of heavy oil and the obtained bitumens. The analysis of bitumens’s components in different fraction ^28^ helps to understand the influence of experimental conditions on the change of the bitumen properties such as softening point, penetration, brittleness point, ductility and the bitumen adhesion on the rock as well as to deduce the possible mechanism of the process. For heavy crude oil, the content of asphaltenes was 6 ± 0.1 wt. %; 21 ± 0.1 wt. % of resins and the combined fractions of saturates and aromatics  of 73 ± 0.3 wt. % were observed. Notably, after the steam distillation process of heavy oil, the results show that, asphaltenes have increased in the obtained product (non-oxidized bitumen) from 6 wt. % up to (11–20 ± 0.1 wt. %), and resins also increased from 21 wt. % to (28–52 ± 0.1 wt. %), while the combined fractions of saturates and aromatics decreased from 73% to a minimum of 56 ± 0.2 wt. %. The composition change of the non-oxidized bitumen occurred due to evaporation, possible cracking and condensation of a light fraction by distillation process which decreases saturates and aromatics fractions in the bitumen residue and relatively increases the high molecular components as (resins and asphaltenes) by condensation^29^. Despite, the increase of high molecular components in the non-oxidized bitumen it is not enough to achieve the required concentration in the expected bitumen of 50/70. Therefore, the steam distillation of heavy oil does not significantly influence on the condensation of aromatics transforming to resins and then to asphaltenes. The results showed that the oxidized bitumen shows different characteristics and properties than that of non-oxidized bitumen. However, after the oxidation process, the asphaltenes increased from 6 wt. % to minimum content of 22% and maximum to 32 ± 0.1 wt. %, the resins from 21 wt. % to 18–28 ± 0.1 wt. % and the mixed fraction of saturates and aromatics decreased from 73 wt. % to 40–53 ± 0.3 wt. % in the oxidized bitumen. The fluctuation of the concentration of resins in the oxidized bitumens took place due to condensation. The saturates and aromatics fractions to resins fraction one side, another to polymerization of resins themselves to asphaltenes^30^. The main reason for the increase of asphaltenes, is the condensation of high molecular components such as di and poly-aromatics in the fractions of resins and aromatics through polymerization reaction converting partially resins to asphaltenes and therefore, increasing the total amount of asphaltenes in the oxidized bitumen^31^. At the same time, the composition of the resins remain unstable concerning the condensation or oxidation of mono-, di- and poly-aromatics because of the influence of temperature, pressure, airflow, and use of nozzles in the system. The oxidation process of hydrocarbon residues can occur at the same time in two ways, by the mechanism of the following reaction^32^: (Eq. 2–Eq. 14)

2 $${\text{RH}} + {\text{O}}_{{2}} \to {\text{ R}}^{ \cdot } + {\text{HOO}}^{ \cdot }$$

The interaction of the formed radicals with a new hydrocarbon molecule leads to product stability:

3 $${\text{R}}^{ \cdot } + {\text{RR}}^{\prime } {\text{H}} \to^{ \cdot } {\text{RR}}^{\prime } {\text{H}}$$

4 $$^{ \cdot } {\text{RR}}^{\prime } {\text{H}} + {\text{R}}^{\prime \prime } {\text{H}} \to^{ \cdot } {\text{RR}}^{\prime } {\text{HR}}^{\prime \prime } {\text{H}}{-}{\text{Disproportion}}.$$

Due to the relatively low concentration of hydrocarbon radicals, their recombination is unlikely, and the interaction of the radicals with oxygen occurs to a lesser extent than with the molecules of the starting material:

5 $${\text{R}}^{ \cdot } + {\text{O}}_{2} \to {\text{ROO}}^{ \cdot }$$

6 $${\text{ROO}}^{ \cdot } + {\text{R}}^{\prime } {\text{H}} \to {\text{ROOH}} + {\text{R}}^{\prime }$$

7 $${\text{ROOH}} \to {\text{RO}}^{ \cdot } { + }^{ \cdot } {\text{OH}}$$

8 $${\text{R}}^{\prime \prime } {\text{H}} +^{ \cdot } {\text{OH}} \to {\text{R}}^{\prime \prime \cdot } + {\text{H}}_{2} {\text{O}}$$

Chain continuation:

9 $${\text{RH}} + {\text{HOO}}^{ \cdot } \to {\text{R}}^{ \cdot } + {\text{H}}_{2} {\text{O}}_{2}$$

10 $${\text{H}}_{2} {\text{O}}_{2} \to 2{\text{OH}}^{ \cdot }$$

11 $${\text{R}}^{\prime } {\text{H}} +^{ \cdot } {\text{OH}} \to {\text{R}}^{\prime \cdot } + {\text{H}}_{2} {\text{O}}$$

Taking the increased softening temperature (t_p_) as the criterion for completion of the reaction and assuming that the concentration of the reacting substance is inversely proportional to the softening temperature, C = a/t_p_ (where a is the proportionality coefficient), the following differential equations are present:

12 $${\text{d}}\left( {{\text{a/t}}_{{\text{p}}} } \right){\text{/d}}\uptau = {\text{K}}_{{\text{o}}} \cdot \ln \left( {{\text{a/t}}_{{\text{p}}} } \right)$$

After differentiation and transformation, we get:

13 $${\text{t}} = \left( {1/{\text{C}}_{{\text{o}}} } \right) \cdot \ln \left( {{\text{t}}_{{{\text{p}}\uptau }} {\text{/t}}_{{{\text{p}}}} } \right)$$

Later the total reaction rate constant (K_o_) is determined by the formula:

14 $${\text{K}}_{{\rm o}} = (1{/}\uptau ) \cdot \ln \left( {{\text{t}}_{{\rm p}{\uptau }} {\text{/t}}_{{{\rm po}}} } \right)$$

where, t_pτ_ is the softening temperature of bitumen during the oxidation time τ; t_po_—the softening temperature of the feedstock.

### Rheological measurement

The rheology of bitumens is presented in Tables 6 and 7. The results indicated that the dynamic viscosity of non-oxidized and oxidized bitumens at 60 °C is 145 and 434 Pa·s, except the dynamic viscosity of the oxide bitumen which is consistent, with the standard STO AVTODOR 2.1–2011 for BNDU 60. On the other hand, results of the kinematic viscosity for both bitumens at 135 °C was 310.8 and 488.4 and it is consistent with the standard STO AVTODOR 2.1-2011 for BNDU 60.

**Table 6:** Rheological measurements and results of the interactions between stone and asphalt of oxidized bitumen.

Properties	Standard for STO AVTODOR 2.1-2011 for BNDU 60	Non-oxidized bitumen
Dynamic viscosity at 60° C, Pa.s	Not less than 300	145 ± 0.1
Viscosity at kinematic at 135° C, mm^2^, s	Not less than 295	310.8 ± 0.1
Viscosity increase coefficient	Not more than 3	2.5
Adhesion to sand according to GOST 6139-2003	full surface coverage	full surface coverage

**Table 7:** Rheological measurements and results of the interactions between stone and asphalt of oxidized bitumen.

Properties	Standard for STO AVTODOR 2.1-2011 for BNDU 60	Oxidized bitumen
Dynamic viscosity at 60° C, Pa.s	Not less than 300	434 ± 0.1
Viscosity at kinematic at 135° C, mm^2^, s	Not less than 295	488.4 ± 0.1
Viscosity increase coefficient	Not more than 3	2
Adhesion to sand according to GOST 6139-2003	full surface coverage	full surface coverage

### Adhesion of the oxidized and non-oxidized bitumen

**Figure 3:**
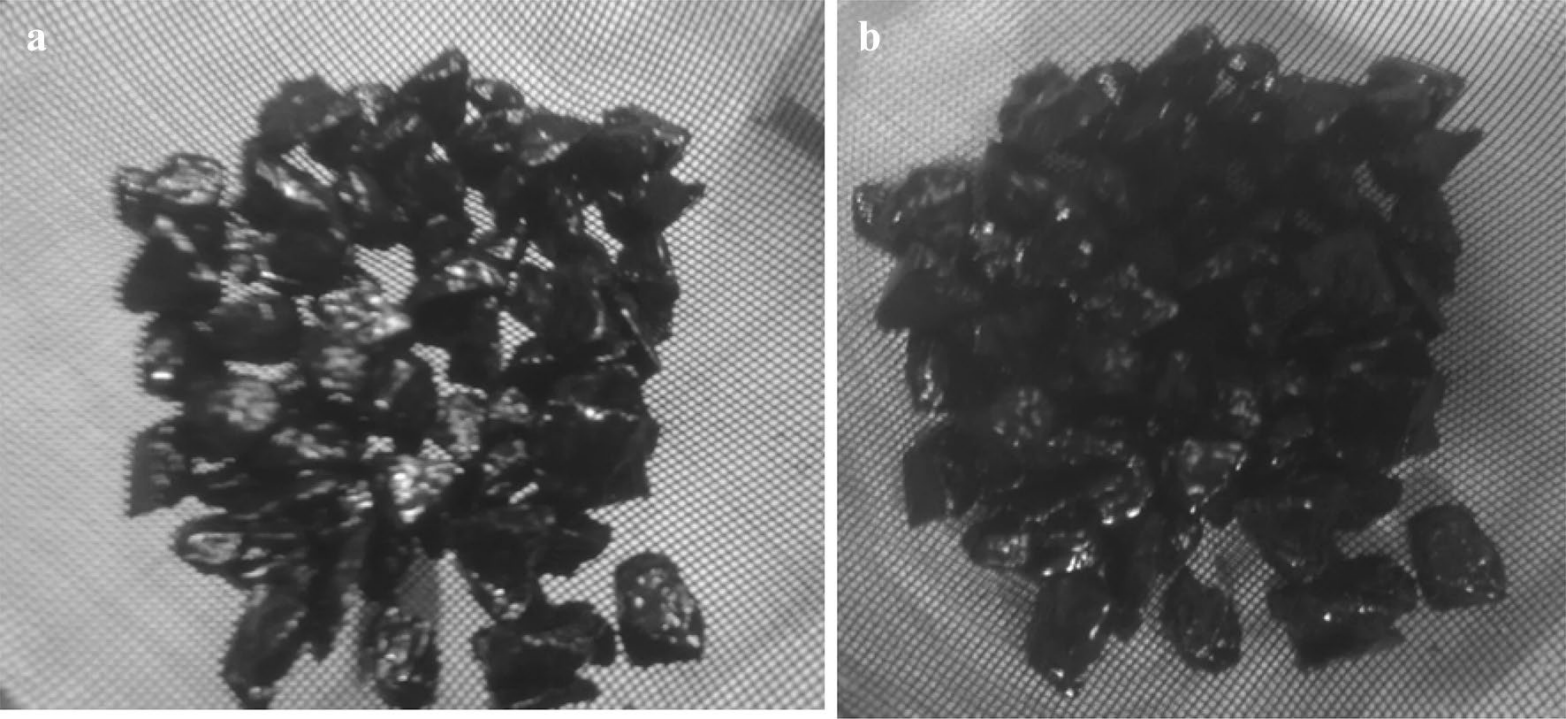
Adhesion of oxidized (**a**) and non-oxidized (**b**) bitumen to acidic rock.

The adhesion properties of the oxidized and non-oxidized bitumen are presented in the Tables 6 and 7. As shown in Fig. 3, the Figure (a) represents the adhesion of oxidized bitumen and Figure (b) of non-oxidized bitumen. So, it should be conclude that both bitumens have good adhesion property to rock surface. Most of the rock surface is fully covered by bitumens mainly that of the oxidized bitumen sample. According to “GOST 1280 s sur 1”, the adhesion of these bitumens to the rock surface presents a "score of 4 ", in another words the binder film completely sticks to the surface.

## Conclusions

In this work, 6 samples of feedstocks were used to produce the road bitumens of BND 50/70 using two different methods. The properties and composition of the obtained bitumens were analyzed.

The results revealed that the obtained oxidized bitumen corresponds to the bitumen BND 50/70, and the properties of this respective bitumen are consistent with the GOST 33133-2014 requirements.

The obtained non-oxidized bitumen using the steam distillation method referred to as commercial bitumen of BND 70/100 with a low softening point of 39 °C and ductility of 0.3 cm at 0 °C.

The non-oxidized bitumens obtained by steam distillation process referred to the bitumens of BND 200/300, BND 100/130, BND 70/100 do not meet the requirements of the current standard, and can be obtained at a temperature above 300 °C and with a reaction time of about 40 min;

According to the Russian standard (GOST 33133-2014), the oxidized bitumen has good results and presents operational properties, and can be used in the practical needs. This bitumen contains 22–32 wt. % of asphaltenes, 18–28 wt. % of resins, and 40–53 wt.% of the mixed fractions of saturated and aromatic;

Finally, it should be noted that, using a packed column in this work has contributed to improving oxidation performance and allowing the production of performant bitumen, reducing the reaction time, and enhancing a good surface contact between airflow and feedstock.

### Experimental section

#### Material

The studied heavy oil sample was taken from the Ashalcha’ oilfield (Tatarstan, Russian Federation) as produced heavy oil by steam-assisted gravity drainage (SAGD) method. In addition, the vacuum residue was obtained as product of vacuum distillation from the same studied heavy oil at temperature and pressure of 390 °C ± 2 °C and 20 mbar, respectively. Table 8 shows the characteristics of heavy oil. The oil has a high density of 985 kg/m^3^ and high viscosity value (2000 cSt). The density of vacuum residue is above 1.00 kg/m^3^, and the viscosity above 2700 cSt. The solvents including benzene (99.96%), hexane (99.98%), isopropyl alcohol (99.95%) were purchased from the local Russian oil-refining company Nizhnekamskneftekhim Ltd to conduct the precipitation, and separation of the initial heavy oil, vacuum residue and the product of their oxidation.

**Table 8:** Properties and characteristics of initial heavy oil.

Sample	Density, kg/m^3^	Viscosity, cSt		Sulfur, %	Water, %	Fractional composition, %		
		20 °C	50 °C			200 °C	300 °C	350 °C
Heavy oil	962 ± 0.1	2742 ± 0.1	366 ± 0.1	4.2 ± 0.1	1	1.1	10.6	9.7

### Methods

#### Steam distillation of heavy oil

**Figure 4:**
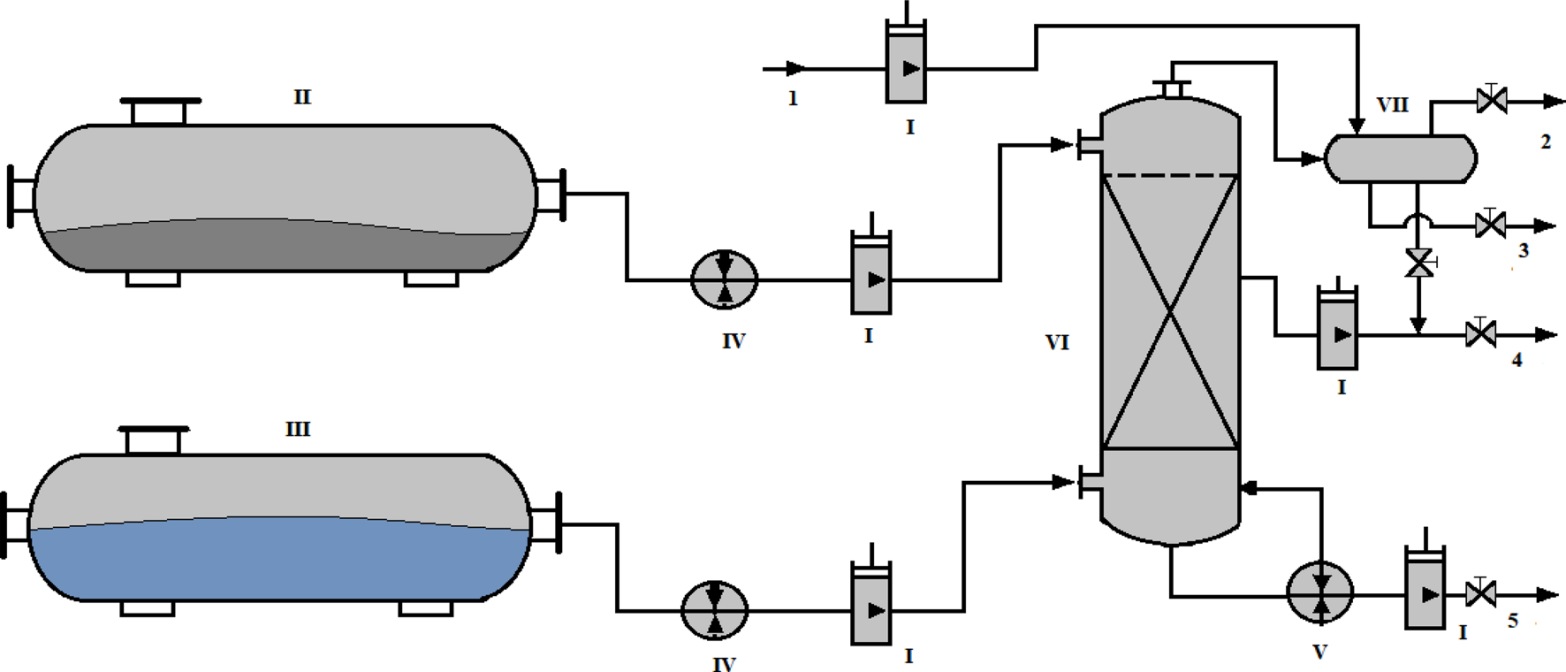
Schematic of the pilot apparatus for steam distillation of heavy oil.

In this paper, the steam distillation process of heavy oil is carried out in continuous mode, vapor/oil ratio varying from (2.6–2.7: 1) at atmospheric pressure. The temperature of steam distillation of heavy oil varied from 300 to 400 °C ± 2 °C and the load capacity of the column was 1 L. The obtained product (heavy residues) was considered as non-oxidized bitumen, and the light products as a water–oil emulsion separated into synthetic oil and water. As shown in Fig. 4, the raw material from tank II was transported through pump I to the heater IV which preheats the heavy oil and routes it to the distillation column V. The superheated steam is supplied to the distillation column from tank III by pump I through a superheater IV. The temperature of the vapor increases in the column using a heating system of the lower part of the column, which makes it possible to heat the hydrocarbons and to separate them into light part (synthetic oil) and the residue (bitumen not oxidized). Also, during the separation through separator VII, the lightest products are collected from the top and the middle of the column, just as gases 2 exit from the top of the separator. The light parts consist of light synthetic oil from separator VII are collected together through a tap valve with heavy synthetic oil 4 and water through pipe 3. At the same time, cold water is supplied by the pump I to cool the products. The mixture of synthetic oil 4 obtained from column VI through the pump I and from condenser VII, and the non-oxidized bitumen 5 is collected through pump I.

#### Oxidation of vacuum residue

**Figure 5:**
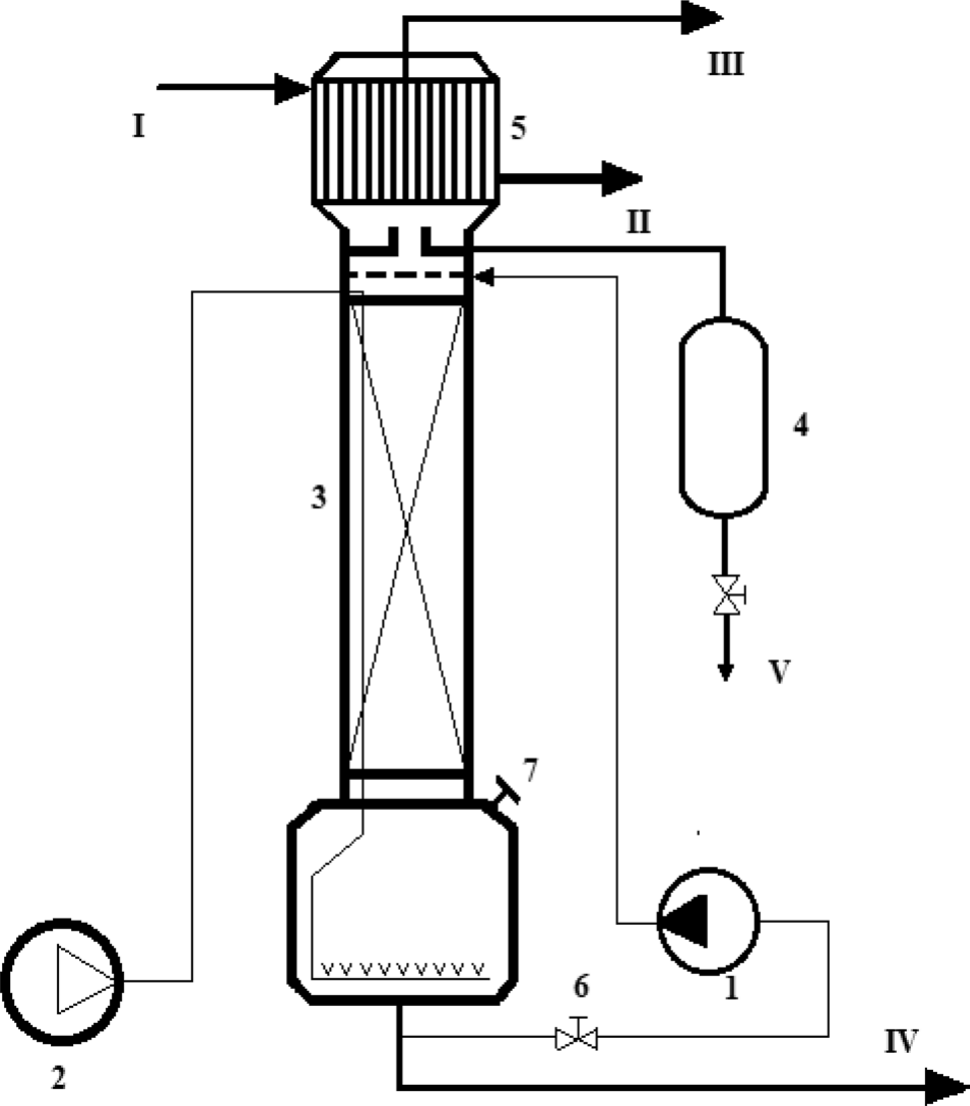
The schematic of the pilot apparatus for the production of oxidized bitumen.

Figure 5 describes the principle of the operational unit of the oxidation process, from which the feedstock (vacuum residue) was and heated to a fluid state and loaded into the bottom of the packed oxidation column 3 through 7. The airflow rate supplied is 5 L/min**·**kg by feedstock. The cube and the column consist of heating devices (tape heat elements) located between the outer walls and the thermal insulation layer, and the temperature inside the column is 220 °C ± 1 °C. After loading the feedstock, heating the cube, and turning on the operating temperature in the circulating column, pump 1 starts and pumps the cube contents to the upper part of the column. Circulating fluid enters the distributor in the column and moves down, providing a counter-current contact with the ascending airflow. Simultaneously with the start-up of pump 1, the air supply to the cube of the column is turned on by compressor 2. Air from the compressor is supplied through a metal tube, a part of which is located under the insulator of the column for heating. The heated air is supplied to the cube of the column 3 through the mother liquor located at the bottom of the column, which evenly distributes the flow over the cross-section of the column. The duration of the process depends on the mass of the loaded vacuum residue, temperature, air flow rate, and brand of bitumen produced. However, in this study, the maximum time of oxidation was 4.15 h. During the oxidation process, an insignificant amount of liquid side-products obtained at the top of the column named black diesel were cooled by condenser 5 and collected through separator V and 4, and the water through II. Oxidation gases consisting of oxides of carbon, sulfur, nitrogen, hydrogen sulfide, unreacted oxygen were evolved through III. The oxidized bitumen is obtained at the bottom of column IV. The non-converted products are recirculated from the column through tap valve 6 and pump I to the top of the column.

#### Bitumen’s group composition analysis

A portion of 1.0 ± 0.1 g of raw material or bitumen was diluted with a 40-fold amount of hexane and thoroughly mixed according to^31, 33^. To completely precipitate the asphaltenes, the solution was left to stand in a dark place for 24 h. The asphaltene precipitate was filtered, transferred to a cartridge of filter paper, and placed in a Soxhlet extractor apparatus to wash the asphaltenes from co-extracted oils and resins. The asphaltenes were washed with hexane until the color of the resulting solvent disappeared. The asphaltenes, washed from the maltenes were washed out of the cartridge with benzene. The solvent was regenerated, and the asphaltenes were brought to constant weight in a vacuum dried at a temperature of 80 °C. After washing the asphaltenes, the filtrate was attached to the base oil and resin solution obtained by filtering the asphaltenes and a part of the hexane so that the dilution of the product with a solvent corresponded approximately to 1:3 ratios. The bitumen fractions are then separated using the chromatographic column; the fractions of the bitumen being (oil extracted using CCl_4_, resins, and asphaltenes). To do this, 60 g of silica gel was poured into the adsorption column (the weight ratio of the deasphaltene oil to silica gel should be approximately 1:40 ml). Silica gel was sealed by tapping the column with a wooden stick, and then soaked with hexane, taken in an amount of 200–250 ml to remove the wetting heat of the adsorbent. After the solvent is fully adsorbed into the silica gel, a sample of the deasphaltenized bitumen or oil is poured into the column. The rate of entry of the product into the sorbent should not exceed 100–120 ml/h. This is achieved by adjusting (using a crane) the rate of selection of the solvent which is impregnated with silica gel. Desorption started 16 h after the starting product was completely adsorbed into silica gel. For desorption of oils, a mixture of benzene-hexane in a volume ratio of 15:85 in an amount of 750 ml, was poured into the column through a separating funnel simultaneously, eluted with a flow rate of 175–200 ml/h began from the bottom of the column. The resin was eluted with an isopropanol-benzene mixture (50:50) in an amount of 200–250 ml. The solvents were distilled off from the fractions on a water bath in a stream of nitrogen. For the final drying of oils and resins, they were transferred to tarred cups and dried to constant weight in a vacuum oven at 70 °C.

#### Standard methods of bitumen’s properties analyses

Determination of the penetration (depth of the needle permeation) was carried out at temperatures of 25 and 0 °C on a PNB-03 penetrometer in accordance with GOST 33136-2014 standards^34^. The analysis of the softening point was carried out on an AKSH-02 device using the Ring and Ball method according to GOST 33142-2014 and EN 1427, ASTM D36, AASHTO T53^35^. The brittleness temperature index was determined by the Fraass method on an ATX-03 device following EN 12593-2013 and GOST 33143-2014, described in^36^. Determination of extensibility (ductility) was carried out at temperatures of 25 and 0 °C according GOST 33138-2014 and EN 13589^37^. Adhesion property of bitumens is obtained using the according to GOST 6139-2003^38^.

#### Initial heavy oil properties analyses

The fractional composition of the heavy oil was determined by the method of atmospheric-vacuum distillation according to GOST 2177-99^39^; density at 20 °C—with an aerometer according to GOST 3900-85^40^; viscosity—according to GOST 6258-85; water content according to GOST 2477-2014^20^; sulfur content according to GOST R 51947-2002^41^.

## References

[1] Nawaz SMN, Alvi S (2018). Energy security for socio-economic and environmental sustainability in Pakistan. Heliyon.

[2] Carré, D. Le Bitume, Un Matériau Pour Le Développement Durable. *RGRA-Revue Generale des Routes et des Aerodromes* **2010**, No. 883, 47

[3] De Castro C, Miguel LJ, Mediavilla M (2009). The role of non conventional oil in the attenuation of peak oil. Energy Policy.

[4] Jayakody S, Gallage C, Ramanujam J (2019). Performance characteristics of recycled concrete aggregate as an unbound pavement material. Heliyon.

[5] Sun F, Yao Y, Li X, Yu P, Ding G, Zou M (2017). The flow and heat transfer characteristics of superheated steam in offshore wells and analysis of superheated steam performance. Comput. Chem. Eng..

[6] Sun F, Yao Y, Chen M, Li X, Zhao L, Meng Y, Sun Z, Zhang T, Feng D (2017). Performance analysis of superheated steam injection for heavy oil recovery and modeling of wellbore heat efficiency. Energy.

[7] Djimasbe, R., Al-muntaser, A. A., Suwaid, M. A., V& arfolomeev, M. A. Comparison of upgrading of heavy oil and vacuum distillation residues by supercritical water. In *IOP Conference Series: Earth and Environmental Science*; IOP Publishing, 2019; Vol. 282, p 12044

[8] Djimasbe R, Ivanov VB, Kemalov AF, Kemalov RA, Valeev TF, Nerobov N (2018). Research of the Technology for the Production of Modified Sulfur Bituminous Binders. Bull. Voronezh State Univ. Eng. Technol..

[9] Sun F, Yao Y, Li X (2018). The heat and mass transfer characteristics of superheated steam coupled with non-condensing gases in horizontal wells with multi-point injection technique. Energy.

[10] Liu P, Mu Z, Li W, Wu Y, Li X (2017). A new mathematical model and experimental validation on foamy-oil flow in developing heavy oil reservoirs. Sci. Rep..

[11] Chengzao J, Zheng M, Zhang Y (2012). Unconventional hydrocarbon resources in china and the prospect of exploration and development. Pet. Explor. Dev..

[12] Allen RG, Little DN, Bhasin A, Glover CJ (2014). The effects of chemical composition on asphalt microstructure and their association to pavement performance. Int. J. Pavement Eng..

[13] Abayev, G. N., Kushnir, Y. V., Dubrovskiy, A. V., Mikhaylova, O. N., Andreyeva, R. A., Dimudu, I. A., & Klyuyev, A. I. Progress in test methods for distillation of petroleum products. *Ind. Serv.* 2013 **48**(3)

[14] Sanei H (2020). Genesis of solid bitumen. Sci. Rep..

[15] Fulcher K, Stacey R, Spencer N (2020). Bitumen from the dead sea in early iron age Nubia. Sci. Rep..

[16] Rudyk S (2018). Relationships between SARA fractions of conventional oil, heavy oil. Natl. Bitumen Residues. Fuel.

[17] Turobova, M. A., Danilov, V. E., & Ayzenshtadt, A. M. Use of wood processing industry waste for bitumen modification. In *IOP Conference Series: Materials Science and Engineering*; IOP Publishing, 2020; Vol. 945, p 12061

[18] Morozov VA, Starov DS, Shakhova NM, Kolobkov VS (2004). Production of paving asphalts from high-wax crude oils. Chem. Technol. Fuels Oils.

[19] Zhang H, Zhu C, Yu J, Tan B, Shi C (2015). Effect of Nano-zinc oxide on ultraviolet aging properties of bitumen with 60/80 penetration grade. Mater. Struct..

[20] Kurlykina, A., Denisov, V., Kuznetsov, D., & Lukash, E. A. Crushed stone-mastic asphalt concrete using sulfur-entraining technologies. *Bulletin of the Belgorod State Technological University. VG Shukhov* **2020**, No. 1

[21] Abdullin AI, Idrisov MR, Emelyanycheva E (2017). Improvement of thermal-oxidative stability of petroleum bitumen using “overoxidation–dilution” technology and introduction of antioxidant additives. Pet. Sci. Technol..

[22] Soenen H, Lu X, Laukkanen O-V (2016). Oxidation of bitumen: Molecular characterization and influence on rheological properties. Rheol. Acta.

[23] Chaala A, Ciochina OG, Roy C (1999). Vacuum pyrolysis of automobile shredder residues: Use of the pyrolytic oil as a modifier for road bitumen. Resour. Conserv. Recycl..

[24] Chaala A, Roy C, Ait-Kadi A (1996). Rheological properties of bitumen modified with pyrolytic carbon black. Fuel.

[25] Shah A, Fishwick R, Wood J, Leeke G, Rigby S, Greaves M (2010). A review of novel techniques for heavy oil and bitumen extraction and upgrading. Energy Environ. Sci..

[26] Loderer C, Partl MN, Poulikakos LD (2018). Effect of crumb rubber production technology on performance of modified bitumen. Constr. Build. Mater..

[27] Mardupenko, A., Grigorov, A., Sinkevich, I., & Tulskaya, A. Technology of modified bitumen production for the road construction. **2019**

[28] Cheraghian G, Wistuba MP (2020). Ultraviolet aging study on bitumen modified by a composite of clay and fumed silica nanoparticles. Sci. Rep..

[29] Kok MV (2001). Thermal investigation of seyitomer oil shale. Thermochim. Acta.

[30] Rishwana SS, Mahendran A, Vijayakumar CT (2015). Studies on structurally different benzoxazines based on diphenols and diamines: Kinetics of thermal degradation and TG-FTIR Studies. Thermochim. Acta.

[31] Savanchuk R (2019). Analysis of the influence of the group composition of oil products on the quality indicators of road bitumen. Almanac World Sci..

[32] Abdullin, A., Idrisov, M., Ganieva, T., & Emelyanycheva, E. *Water-bitumen Emulsions*; Litres, 2017

[33] Volkov, V. Y., Al-Muntaser, A. A., Varfolomeev, M. A., Khasanova, N. M., Sakharov, B. V., Suwaid, M. A., Djimasbe, R., Galeev, R. I., & Nurgaliev, D. K. Low-field NMR-relaxometry as fast and simple technique for in-situ determination of SARA-composition of crude oils. *J. Pet. Sci. Eng. 196*, 107990

[34] Yadykina, V. V., Akimov, A. E., Trautvain, A. I., & Kholopov, V. S. Influence of DAD-TA temperature-reducing additive on physical and mechanical properties of bitumen and compaction of asphalt concrete. In *IOP Conference Series: Materials Science and Engineering*; IOP Publishing, 2018; Vol. 327, p 32006.

[35] Moshref, H. S., Kutyin, Y. A., & Telyashev, E. G. Petroleum road bitumen. Standards, quality, technology, prospects. *Oil & Gas Business* **2012**, No. 6

[36] Kemalov, A. F., Kemalov, R. A., Abdrafikova, I. M., Fakhretdinov, P. S., & Valiev, D. Z. Polyfunctional modifiers for bitumen and bituminous materials with high performance. *Advances in Materials Science and Engineering* **2018**, *2018*

[37] Pereira L, Freire AC, da Costa MS, Antunes V, Quaresma L, Micaelo R (2018). Experimental study of the effect of filler on the ductility of filler-bitumen mastics. Constr. Build. Mater..

[38] Zhang J, Airey GD, Grenfell JRA (2016). Experimental evaluation of cohesive and adhesive bond strength and fracture energy of bitumen-aggregate systems. Mater. Struct..

[39] Khamidullin, R., Kovalchuk, D., & Galiullin, E. A. Experimental studies of the distillation of heavy oil residues in an inert gas environment. *Kazan Technol. Univ. Bull.* **2015**, *18*(20).

[40] Zabrodin, A., Alibekov, S. Y., Zabrodina, N., Salmanov, R., & Maryashev, A. Analysis of the physical and mechanical properties of M100 fuel oil. *Kazan Technological University Bulletin* **2013**, *16* (7).

[41] Dedov, A. G., Marchenko, D. Y., Zrelova, L. V., Ivanova, E. A., Sandzhieva, D. A., Parkhomenko, A. A., Budinov, S. V., Lobakova, E. S., & Dol’nikova, G. A. New method for determination of total of organic sulfur compounds in hydrocarbon media. *Pet. Chem.* **2018**, *58*(8), 714–720.

[42] Homayuni F, Hamidi AA, Vatani A (2012). An experimental investigation of viscosity reduction for pipeline transportation of heavy and extra-heavy crude oils. Pet. Sci. Technol..

